# *Xenopus *NM23-X4 regulates retinal gliogenesis through interaction with p27Xic1

**DOI:** 10.1186/1749-8104-4-1

**Published:** 2009-01-05

**Authors:** Toshiaki Mochizuki, Aikaterini Bilitou, Caroline T Waters, Kamran Hussain, Massimo Zollo, Shin-ichi Ohnuma

**Affiliations:** 1 Hutchison/MRC Research Centre, Department of Oncology, University of Cambridge, Hills Road, Cambridge CB2 0XZ, UK; 2 University of Biotechnological Sciences, Federico II, Naples, Via Comunale Margherita 482, 80145, Napoli, Italy; 3 UCL Institute of Ophthalmology, University College London, 11-43 Bath Street, London, EC1V 9EL, UK

## Abstract

**Background:**

In *Xenopus *retinogenesis, p27Xic1, a *Xenopus *cyclin dependent kinase inhibitor, functions as a cell fate determinant in both gliogenesis and neurogenesis in a context dependent manner. This activity is essential for co-ordination of determination and cell cycle regulation. However, very little is known about the mechanism regulating the context dependent choice between gliogenesis versus neurogenesis.

**Results:**

We have identified NM23-X4, a NM23 family member, as a binding partner of p27Xic1. NM23-X4 is expressed at the periphery of the ciliary marginal zone of the *Xenopus *retina and the expression overlaps with p27Xic1 at the central side. Our *in vivo *functional analysis in *Xenopus *retina has shown that knockdown of NM23-X4 activates gliogenesis. Furthermore, co-overexpression of NM23-X4 with p27Xic1 results in the inhibition of p27Xic1-mediated gliogenesis, through direct interaction of NM23-X4 with the amino-terminal side of p27Xic1. This inhibitory effect on gliogenesis requires serine-150 and histidine-148, which correspond to the important residues for the kinase activities of NM23 family members.

**Conclusion:**

This study demonstrates that NM23-X4 functions as an inhibitor of p27Xic1-mediated gliogenesis in *Xenopus *retina and suggests that this activity contributes to the proper spatio-temporal regulation of gliogenesis.

## Background

Co-ordination of cell cycle regulation and developmental signaling is essential for proper development of the nervous system [1,2]. Recently, it has become clear that developmental signals can act on cell cycle regulation of neural progenitors, while cell cycle components can influence developmental signals. However, little is known about the detailed mechanisms by which this co-ordination occurs. The retina is an ideal system to analyze the communication between cell cycle regulation and cell fate determination. It consists of six major types of neurons: retinal ganglion cells – horizontal, amacrine, bipolar, cone and rod photoreceptor cells; and one glial cell type – Müller glial cells. All neurons and glial cells are generally produced from a multipotent pool of retinal stem cells in a sequentially determined order [3,4]. In the *Xenopus *retina, retinal ganglion cells are the first cell type to be determined, at around stage 27/28, while bipolar and Müller glial cells are the last to be determined at around stage 37/38.

In the retina, differentiation of neurons and glial cells is preceded by cell cycle exit of the progenitors during early G1 phase. The fate of each cell type appears to be influenced during the final cell cycle [5-8]. Insufficient accumulation of a cell fate determinant in the last cell cycle allows cell cycle progression without determination [9]. G1-phase cell cycle components, such as cyclin Ds, have important roles in the choice between continuous proliferation and cell fate determination [10]. Cells arrested in early G1 are more likely to be influenced by determinants than cycling cells, while cell cycle activation tends to inhibit the activity of determinants [8]. In addition, many determinants have the ability to induce cell cycle exit in G1 [2,11,12], suggesting bi-directional communication between cell cycle components and determinants.

p27Xic1 is a *Xenopus *member of the Cip/Kip cyclin dependent kinase (CDK) inhibitor (CDKI) family that promotes Müller glial cell fate determination in the retina [13]. The Cip/Kip CDKI family has a conserved CDK/cyclin binding domain in its amino terminus and member-specific sequences in the carboxyl terminus. p27Xic1 activates gliogenesis through its amino terminus but in a cell cycle regulation-independent manner. However, in the presence of proneural genes, such as *Xath5*, p27Xic1 potentiates the activities of proneural genes [8]. A similar neurogenic activity of p27Xic1 has been demonstrated in *Xenopus *primary neurogenesis [14,15] and is also mediated through the amino terminus of the protein. Also, p57Kip2 influences specification of amacrine cell subtypes in the mouse retina [16]. All these observations indicate that the Cip/Kip CDKIs regulate both gliogenesis and neurogenesis through their amino termini in a context-dependent manner. Recently, this dual role has been demonstrated in the mammalian system as well. Mouse retinal progenitors lacking Math5, a determinant of retinal ganglion cells, upregulate p27Kip1 and differentiate into Müller glial cells [17]. Furthermore, CDKIs regulate differentiation processes through regulation of actin and microtubule structure [18-22]. These activities are mediated by direct interaction between the carboxyl termini of CDKIs and the regulators of cytoskeletal structures. These observations indicate that CDKIs have three independent activities: cell cycle regulation, cell fate determination, and regulation of cytoskeletal structures. Previous work has shown that the amino terminus of p27Xic1 promotes neurogenesis by stabilizing neurogenin [14], but this mechanism cannot explain how p27Xic1 promotes glial rather than neuronal fate in the retina. To address this conflict, we have screened for proteins that interact with the amino terminus of p27Xic1 and regulate its gliogenic activity in the *Xenopus *retina. Here, we identify NM23-X4 as a binding partner of p27Xic1 and show that NM23-X4 regulates gliogenic activity of p27Xic1 in *Xenopus *retinogenesis.

## Results

### Identification of NM23-X4 as a novel binding protein of p27Xic1

**Figure 1:**
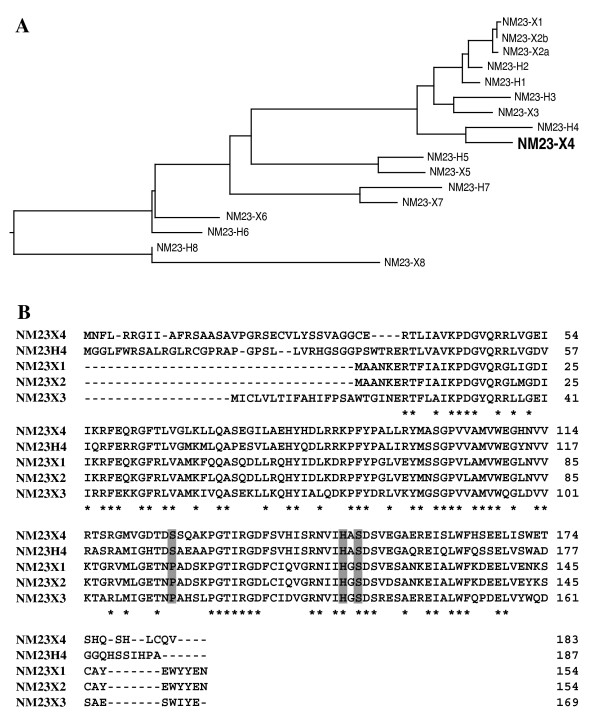
**Sequence alignment of NM23-X4**. **(A) **Phylogenetic tree of human and *Xenopus *NM23 members. The tree was constructed using the Clustal W method with the parameter PAM250. **(B) **Nucleotide sequence alignment of NM23-X4 with NM23-H4, -X1, -X2, and -X3. Asterisks indicate identical amino acids among all NM23 family members. The previously reported important amino acid residues are shown as shaded boxes.

To identify proteins that interact with the amino terminus of p27Xic1, we performed bacterial two-hybrid screening using a *Xenopus *two-hybrid library (3 × 10^6 ^clones) and the amino terminus (1–96 amino acids) of p27Xic1 as bait. One of the positive hits encodes a protein sequence with high homology to NM23 proteins. The NM23 family consists of nucleotide diphosphate kinases (NDPKs), which exchange γ-phosphate between nucleotide triphosphate and diphosphate [23]. Ten members are known to comprise the human family (NM23-H1 to -H9 and RP2). Apart from NDPK activity, NM23-H1 and -H2 have been shown to possess additional activities, such as serine/threonine kinase, histidine dependent kinase, transcription factor, 3'-5' exonuclease, and apoptosis induced DNase activities (for reviews, see [24,25]). Although mouse NM23-M1 is highly expressed in the central nervous system [26], very little is known about the role of NM23 members in neurogenesis and gliogenesis. We have identified five novel *Xenopus *members of the NM23 family by searching the *Xenopus *EST database in addition to three already known NM23 members [27]. Sequence alignment revealed that our identified clone has the highest homology with the human NM23-H4 and we thus named it NM23-X4 (Figure 1A–B).

### Interaction between NM23 and CDKIs

**Figure 2:**
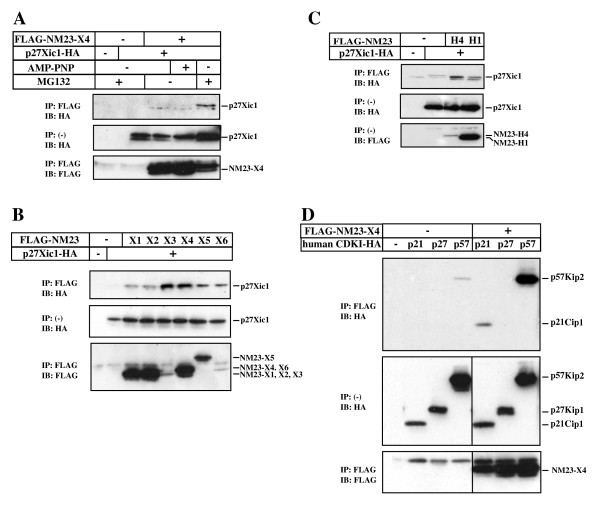
**Interaction between NM23 and CDKIs**. **(A) **Co-immunoprecipitation of NM23-X4 and p27Xic1 in COS7 cells. p27Xic1 interacts strongly with NM23-X4 in the presence of the proteasome inhibitor MG132. **(B) **Interaction of p27Xic1 with NM23-X1, -X2, -X3, -X4, -X5, or -X6. **(C) **Interaction of p27Xic1 with the human NM23-H1 and -H4 homologs. **(D) **NM23-X4 interacts strongly with human p21Cip1 and p57Kip2, but not with p27Kip1. All analyses were performed in COS7 cells in the presence of MG132 unless otherwise stated.

Identification of NM23-X4 as a binding partner of p27Xic1 by two-hybrid screening prompted us to verify this interaction in cells. We were able to confirm this protein-protein interaction by immunoprecipitation after transfection of tagged expression constructs in COS7 cells. Initially, as shown in Figure 2A, immunoprecipitation of NM23-X4 with p27Xic1 produced an extremely weak band (lane 3). Since p27Xic1 is an unstable protein, which is degraded by the proteasome [28], we analyzed the interaction in the presence of a proteasome inhibitor MG132. Lane 5 shows a strong band corresponding to p27Xic1, indicating that indeed NM23-X4 binds to p27Xic1. Also, we analyzed the effect of AMP-PNP, a non-hydrolysable analogue of ATP that stabilizes interactions between NM23-H1 and the kinase suppressor of Ras [29]. However, AMP-PNP did not have any effect on p27Xic1 interaction with NM23-X4. All the following immunoprecipitation experiments were performed in the presence of MG132 unless otherwise stated.

Next, we wanted to know if p27Xic1 interacts with other NM23 family members. As shown in Figure 2B, NM23-X3 also binds strongly to p27Xic1, while all other NM23s tested interacted weakly. The human NM23-H1 and H4 interacted with p27Xic1 similarly to their *Xenopus *orthologs (Figure 2C). Taken together, these results suggest that NM23-X4 and/or X3 are likely to be biologically significant binding partners of p27Xic1.

Mammals have three Cip/Kip CDKIs (p21Cip1, p27Kip1, and p57Kip2). Interestingly, NM23-X4 interacted with p21Cip1 and p57Kip2, but not with the human p27Kip1 (Figure 2D). We identified two other *Xenopus *CDKIs, p16Xic2 and p17Xic3, which also have gliogenic functions, similar to that of p27Xic1, although their expression levels in the developing retina are lower [30]. Interestingly, we found that p16Xic2 does not interact with NM23-X4 (unpublished data). Sequence alignment revealed that p27Xic1 is likely to be an orthologue of p57Kip2 [30]. Since NM23-X4 is a binding partner of p27Xic1/p57Kip2, we reasoned that NM23-X4 is likely to work upstream of, or at the same level with, p27Xic1 as an effector in retinogenesis.

**Figure 3:**
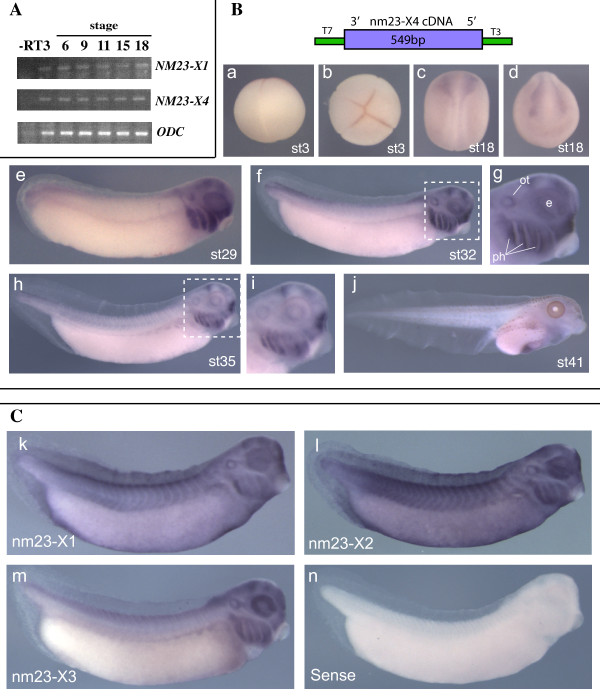
**Expression of NM23-X4 and other *Xenopus *family members in embryogenesis. (A) **Semi-quantitative RT-PCR analysis of NM23-X4 and NM23-X1 at stages 6, 9, 11, 15 and 18 of embryogenesis. Ornithine decarboxylase (ODC) was used as an internal control. **(B) **Whole-mount *in situ *hybridization of NM23-X4 with a probe containing the cDNA cloned in pBluescript as shown in the schematic. Expression is not detected until after gastrulation at neurula stages and with persistence in the head region. Shown are: (a) stage 3, lateral view, animal up; (b) stage 3, animal view (c) stage 18, dorsal view, anterior up; (d) stage 18, anterior view, dorsal up; (e) stage 29, dorsal up, anterior right; (f) stage 32, with enlarged view marked in dashed line and shown in (g); (h) stage 35, with enlarged view marked in dashed line and shown in (i); (j) stage 41. ot, otic vesicle; ph, pharyngeal arches; e, eye. **(C) **Whole-mount *in situ *hybridization of NM23-X1, -X2 and -X3 at representative tadpole stage 33: (k) NM23-X1 and (l) NM23-X2 are ubiquitously expressed, while (m) NM23-X3 is localized in the head region. Sense control is shown in (n).

NM23-X4 and -X3 are expressed in the ciliary marginal zone of the retinaThe expression patterns of the novel NM23 family members were analyzed in *Xenopus *embryos. Semi-quantitative RT-PCR analysis showed that there are significant levels of maternal and zygotic NM23-X4 mRNA in early embryos (Figure 3A). However, in whole mount *in situ *hybridization, specific localized staining was not observed until neurula stages (Figure 3B, a–b), suggesting that before gastrulation NM23-X4 is uniformly expressed in low levels. In neurula, NM23-X4 expression is detected in the neural fold with staining in the eye primordia (Figure 3B, c–d). At later stages, its expression is restricted to the head area, with stronger staining in the eye, otic vesicle, brain, spinal cord, pharyngeal arch, and pronephros (Figure 3B, e–j). We also analyzed the expression patterns of other NM23 family members. All NM23 family members are expressed in the central nervous system, although their expression pattern in other tissues varies (data not shown). NM23-X1 and -X2 show ubiquitous expression (Figure 3C, k–l), while NM23-X3, like NM23-X4, shows prominent staining in the head region (Figure 3C, m).

**Figure 4:**
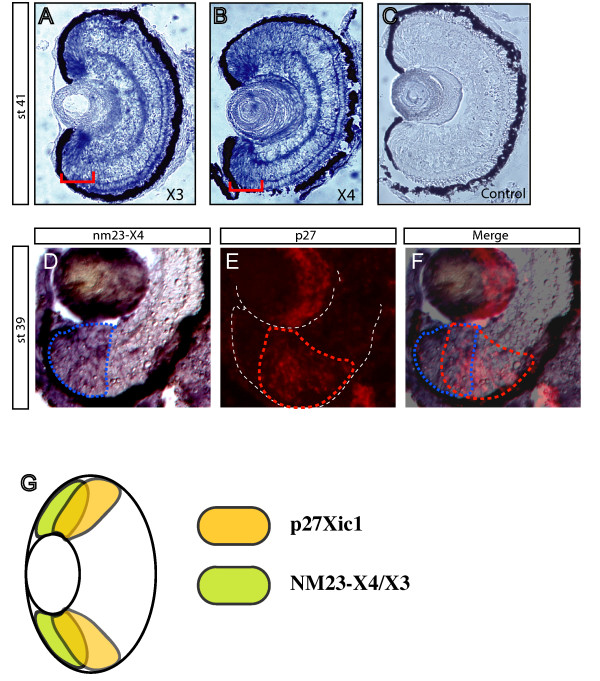
**Expression pattern of NM23-X3, -X4 and p27Xic1 in *Xenopus *retina. (A, B) **Both NM23-X3 (A) and NM23-X4 (B) are expressed in the CMZ of *Xenopus *retina as shown by *in situ *hybridization on stage 41 cryosections. Red brackets indicate the expressed regions in the CMZ. **(C) **Sense-probe used as negative control. **(D, E) **NM23-X4 expression pattern in the retinal CMZ (D) overlaps with that of p27 (E), as underlined by dotted lines in blue and red, respectively. *In situ *hybridization was done at stage 39 sequential cryosections with signal visualized with BM purple and Fast Red, respectively. **(E) **Merged image; white dotted line marks the retinal epithelium boundaries. **(G) **A schematic diagram of expression of p27Xic1 and NM23-X4 or -X3 in embryonic *Xenopus *retina.

The *Xenopus *retina continuously grows during the entire life of the animal. At late stages, new cells are produced from the peripheral region of the retina, the ciliary marginal zone (CMZ). Retinal stem cells are located at the most peripheral region of the CMZ and gradually generate all types of neurons and glial cells towards the most central part. Therefore, genes important for cell fate determination are expressed in the most central part of the CMZ, whilst genes related to retinal stem cell function are expressed in the most peripheral region [8,31]. We analyzed the expression patterns of NM23 members in the retina by *in situ *hybridization on tissue sections. At stage 41, the expression of NM23-X4 and -X3 persists in the CMZ (Figure 4A–C), whilst other NM23 members are ubiquitously expressed in the retina (data not shown). This suggests that NM23-X4 and -X3 may be involved in cell fate determination during retinogenesis. More precise analysis was performed using earlier stages when the CMZ covers a much wider area than that at stage 41. Interestingly, the expressed domain of NM23-X4 overlaps with that of p27Xic1 at the central CMZ, but extended to a more peripheral region than p27Xic1 (Figure 4D–F). The same pattern is seen with NM23-X3, while expression of NM23-X1 was ubiquitous (unpublished data).

### NM23-X4 is a negative regulator of p27Xic1-mediated gliogenesis

**Figure 5:**
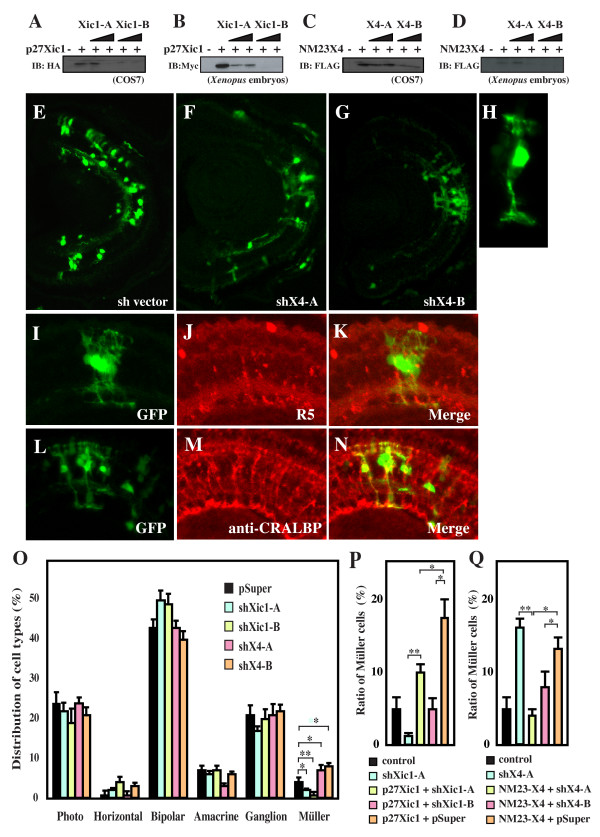
**Reduction of NM23-X4 increases the Müller glial cell population. (A-D) **shRNAs against p27Xic1 and NM23-X4 are efficient in knocking down their respective protein expression in cell culture and *Xenopus *embryos. The indicated short hairpin RNA (shRNA) constructs and the corresponding tagged expression constructs were co-transfected in COS7 cells and co-injected in two-cell stage *Xenopus *embryos. The effect was analyzed by immunoprecipitation of total lysate of cells or embryos (see Materials and methods). **(E-G) **Stage 41 retinal section after transfecting with *pSuper *vector and *GFP *(E), with *shX4-A *and *GFP *(F), or with *shX4-B *and *GFP *(G). **(H) **Enlarged view of a green fluorescent protein (GFP)-positive Müller glial cell transfected with *shX4-B*. **(I-K) **Staining against the Müller glial marker R5 at stage 41 retina transfected with *shX4-B*: (I) GFP; (J) R5 staining; (K) merged view. **(L-N) **Anti-CRALBP staining of Müller glial cells at stage 41 retina transfected with *shX4-B*: (L) GFP, (M) anti-CRALBP, (N) merged view. **(O) **Cell type distribution in the stage 41 retina transfected with the indicated construct plus *GFP *expressed in percentages. **(P-Q) **Rescue of the knock-down effect in the cell type distribution in the retina by co-introduction of *shRNA *construct with p27Xic1 (P) and NM23X4 (Q) expression constructs. The Müller glial cell percentages for each condition are shown. Single and double asterisks correspond to *P *≤ 0.05 and 0.01, respectively; error bars indicate standard error of the mean.

Our findings of the overlapping expression patterns and protein interaction suggest that NM23-X4 functionally interacts with p27Xic1 in retinogenesis. To further elucidate this, we took a loss-of-function approach using short hairpin RNA (shRNA) constructs. The small interfering RNA/shRNA-based system has been previously reported by several groups [32-35]. We first tested the efficiency of the approach using two shRNA constructs targeted against p27Xic1 (*shXic1-A *and *shXic1-B*). Figure 5A,B show that both *shXic1-A *and -*B *reduced p27Xic1 levels in both cell cultures and *Xenopus *embryos. These plasmids were co-lipofected with GFP as a tracer into *Xenopus *eye primordia at stage 15. The effect on distribution of retinal cell types was analyzed at stage 41 by counting the numbers of differentiated neurons and Müller glial cells. Both *shXic1-A *and -*B *significantly reduced the proportion of Müller glial cells more effectively than a previously tested plasmid producing antisense p27Xic1 RNA (Figure 5O and data not shown) [13]. Furthermore, these effects were rescued by co-overexpression with p27Xic1 (Figure 5P). We then designed two shRNA constructs against NM23-X4 (*shX4-A *and *shX4-B*) that recognize different target sequences and analyzed their effects on retinogenesis. When the shRNA constructs were introduced with tagged NM23-X4 in cells or *Xenopus *embryos, both shRNA constructs reduced the exogenous NM23-X4 protein levels, albeit with slightly different efficiencies (Figure 5C,D). Due to lack of an antibody specific for *Xenopus *NM23-X4, it was not possible to check the effect on endogenous protein levels. Monitoring the mRNA levels was also difficult because of the low efficiency of lipofection in the retina. Interestingly, both lipofections of *shX4-A *and -*B *caused a two- to three-fold increase in the number of cells with the morphology of Müller glial cells, which have cell bodies residing in the inner nuclear layer with a complex process expanding from the apical side to the basal side of the retina (Figure 5E–H). The cell identity was confirmed by staining for known Müller glial cell markers using the R5 antibody, which stains the endfeet of Müller cells (Figure 5I–K), and anti-CRALBP, which stains the processes of Müller cells (Figure 5L–N). We further verified the phenotype by the lack of staining for markers of other cell types (data not shown). As shown in Figure 5O, both *shX4-A *and -*B *significantly increased the proportion of Müller glial cells. To confirm the specificity of shRNAs, a rescue experiment was performed (Figure 5Q). The effects of both *shX4s *were rescued by co-introduction of a NM23-X4 expression construct. These results indicate that endogenous NM23-X4 functions as a negative regulator of gliogenesis and suggests that NM23-X4 may downregulate the p27Xic1-mediated gliogenesis.

### NM23-X4 inhibits p27Xic1-mediated gliogenesis through their direct interaction

**Figure 6:**
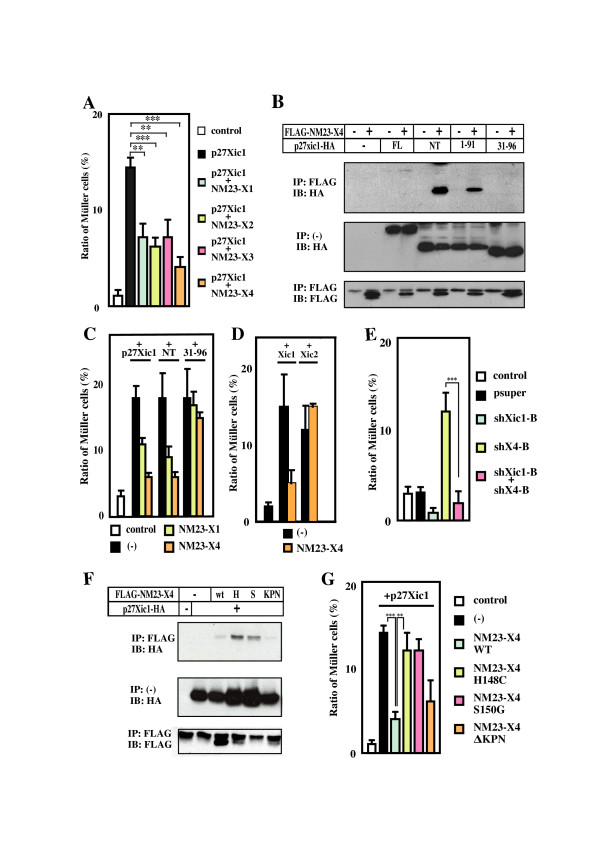
**NM23 inhibits p27Xic1-mediated gliogenesis through their interaction**. **(A) **Co-expression of a NM23 member with p27Xic1 inhibits p27Xic1-mediated gliogenesis. **(B) **Interaction of NM23-X4 with deleted versions of p27Xic1. Full length, amino-terminal NT(1–96), and 1–91 portions of p27Xic1 interact with NM23-X4, but the 31–96 portion does not. **(C) **The interaction between p27Xic1 and NM23 is responsible for the inhibitory function of NM23 on Müller glial cell phenotype. NM23-X4 blocked glial induction by interacting with the amino-terminal and 1–91 portions of p27Xic1 but not with the 31–96 portion. **(D) **NM23-X4 cannot inhibit gliogenesis mediated by p16Xic2. Müller glial cell percentage in the retina after co-introduction of NM23-X4 and Xic1 or Xic2. **(E) **Effect of co-introduction of *shX4-B *and -*B *constructs in the retina. Activation of gliogenesis by *shX4*-B requires p27Xic1. **(F) **Interaction of p27Xic1 with mutants of NM23-X4. Wild type (wt), H148C (H), S150G (S) and ΔKPN were tested for their interaction with p27Xic1. **(G) **Wild type and the ΔKPN blocked Müller cell induction by p27Xic1, but H148C and S150G did not. Double and triple asterisks correspond to *P *≤ 0.01, and 0.001, respectively; error bars indicate standard error of the mean.

Our results suggest that NM23-X4 negatively regulates p27Xic1 activity. In order to test this hypothesis, we co-overexpressed p27Xic1 and NM23-X4 in the retina by lipofection and analyzed the effect on gliogenesis. As reported previously, p27Xic1 strongly activates gliogenesis (Figure 6A). Interestingly, co-expression with NM23-X4 strongly inhibited this effect (Figure 6A). Other NM23 family members, NM23-X1, -X2, and -X3, also inhibited p27Xic1-mediated gliogenesis, although the inhibition was weaker than that mediated by NM23-X4.

In order to gain more insight into the NM23-X4 action, we examined the putative NM23-X4 interaction domain of p27Xic1. Mutational analysis showed that NM23-X4 binds to two amino-terminal constructs of p27Xic1, NT (1–96) and 1–91, but not to the carboxy-terminal one (Figure 6B and data not shown). Interestingly, NM23-X4 did not interact with the 1–30 construct or the 31–96 construct, which is sufficient for both cyclin/CDK binding and Müller cell inductive activity similarly to NT and 1–91 constructs [13], suggesting that NM23-X4 binding requires an area covering both parts of 1–30 and 31–91. Also, the realization that the immunostained bands of NT and 1–91 are always stronger than that of the wild type suggested that the carboxy-terminal region of p27Xic1 might negatively influence the binding between NM23-X4 and p27Xic1. We then analyzed the effect of NM23-X4 and -X1 on gliogenesis mediated by p27Xic1 derivatives (Figure 6C). Interestingly, both NM23-X4 and -X1 effectively inhibited the glial-promoting effect of full length and NT constructs, while the effects of the 31–96 construct were hardly inhibited by these isoforms. These results imply that the inhibitory function of NM23 proteins requires their interaction with p27Xic1 and that NM23-X4 does not inhibit p27Xic1 activity downstream of p27Xic1. Consistent with this, NM23-X4 does not inhibit gliogenesis mediated by p16Xic2 (Figure 6D), with which it does not bind. Next, the effect of co-introduction of *shX4-B *and -*B *on gliogenesis was analyzed. Figure 6E shows that *shX4-B *does not activate gliogenesis when p27Xic1 is downregulated (in the presence of *shXic1-B)*, indicating that activation of gliogenesis by *shX4-B *occurs in the presence of p27Xic1.

### Serine-150 and histidine-148 are required for the NM23-X4 activity

We then tried to identify the residue(s) or domain(s) of the NM23 proteins important for the regulation of p27Xic1 activity. Some critical residues and domains of NM23-H1 have been identified previously (Figure 1B). Histidine-118 of NM23-H1 (histidine-148 of NM23-X4) is required for the NDPK and histidine-dependent kinase activities [36]. Serine-120 of NM23-H1 (serine-150 of NM23-X4) is also important for histidine dependent kinase activity [36]. The KPN loop forms part of the active center important for the kinase activity and mediates hexamer formation [37]. Therefore, three mutants of NM23-X4 (NM23-X4 H148C, S150G, and ΔKPN) were constructed and their interaction with p27Xic1 and effects on p27Xic1-mediated gliogenesis were analyzed. As shown in Figure 6F, all three mutants interacted with p27Xic1. Interestingly, NM23-X4 H148C and S150G mutants did not inhibit p27Xic1-mediated gliogenesis, while ΔKPN did (Figure 6G), suggesting that NDPK and/or histidine dependent protein kinase activities are required for regulation of p27Xic1 activity. This might also suggest that deletion of the KPN loop in the ΔKPN mutant does not structurally abrogate the active center formation as is reported for the case of NM23-H1 [38].

### Overexpression of NM23 family members promotes gliogenesis

**Figure 7:**
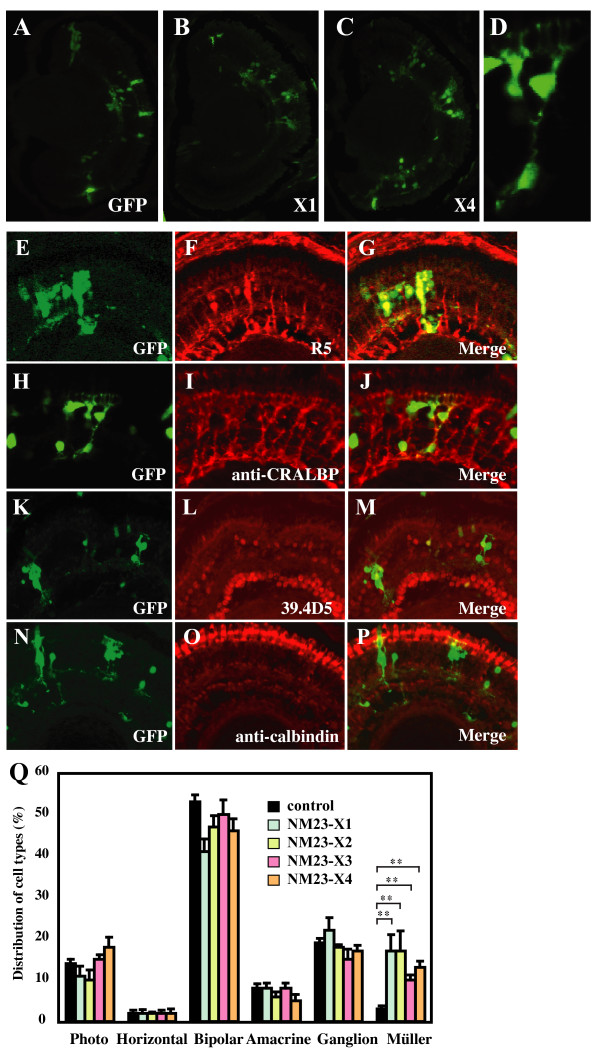
**Overexpression of NM23 activates gliogenesis. (A-D) **Retinal section at stage 41 after lipofection with *GFP *(A), *NM23-X1 *(B), or *NM23-X4 *(C). A representative image of Müller glial cells in the retina transfected with *NM23-X4 *and *GFP *(D). **(E-P) **Immunostaining of NM23-X4 lipofected retinas with cell specific markers. (E-G) Müller glia staining using R5 antibody: (E) green fluorescent protein (GFP); (F) R5; (G) merged. (H-J) anti-CRALBP staining of Müller glial cells: (H) GFP; (I) anti-CRALBP; (J) merged. (K-M) Ganglion cell staining against islet-1 using 39.4D5 antibody: (K) GFP; (L) 39.4D5 staining; (M) merged. (N-P) Photoreceptor staining with anti-calbindin: (N) GFP; (O) calbindin staining; (P) merged. **(Q) **Distribution of cell types in the retina after lipofection with NM23 family members.

Overexpression of NM23-X4 alone in the retina increased the proportion of Müller cells (Figure 7A–D). Staining against the Müller glial markers R5 and CRALBP verified the Müller cell identity of NM23-X4 expressing cells that presented a glial cell morphology (Figure 7E–J). Anti-calbindin and 39.4D5 staining was performed to visualize photoreceptor and ganglion cells in order to verify the phenotype and confirm the cell type percentages (Figure 7K–P). Further analysis revealed that overexpression of NM23-X1, -X2, -X3, and -H1 also induced similar gliogenic phenotype (Figure 7Q and data not shown). Since loss of function causes an increase in the Müller cell population, one would expect that overexpression of NM23 would have the opposite effect. Possibly, these observations show that NM23s function in a context-dependent manner to regulate neurogenesis versus gliogenesis. Furthermore, it has been observed that the glial-promoting activity of NM23-X4 is not affected by loss of function of p27Xic1 (data not shown), suggesting that the activation of gliogenesis by NM23 does not require p27Xic1. This implies that NM23-X4 might have a second mechanism of action, independent of the suppression of p27Xic1-mediated gliogenesis. Ongoing experiments will further elucidate this.

### Effect of NM23-X4 on cell cycle regulation

**Figure 8:**
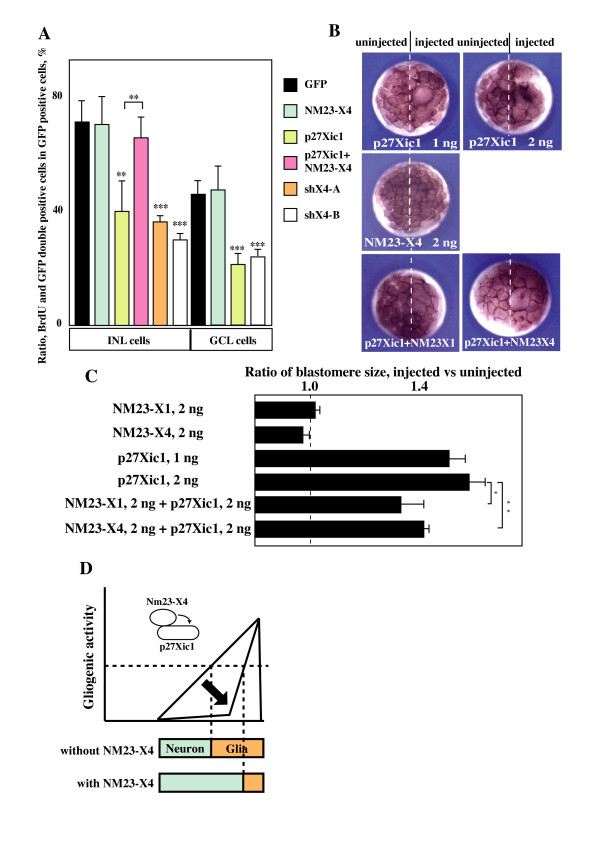
**Effect of NM23-X4 on cell cycle regulation**. **(A) **BrdU assay in *Xenopus *retina. The indicated constructs were co-lipofected with GFP at stage 15. BrdU was injected into embryos from stage 30 to 41 and ratios of BrdU positive cells within GFP positive lipofected cells in the inner nuclear layer (INL) and ganglion cell layer (GCL) were determined as described in the Materials and methods. *NM23*-X4 does not influence proliferation, while *shX4-A *and -*B *reduce the ratio of BrdU positive cells. Co-overexpression of NM23-X4 with p27Xic1 inhibits p27Xic1 mediated cell cycle arrest. **(B, C) **NM23 can inhibit p27Xic1-mediated cell cycle arrest in early embryos. After mRNA injection into a two-cell stage blastomere, the blastomere size of the injected side was compared with the uninjected side at stage 7. The effect was evaluated and the data are presented in the graph shown in (C). **(D) **A proposed model for NM23-X4 function in retinal cell fate determination. Both NM23-X4 and p27Xic1 are expressed in the CMZ. NM23-X4 expression starts first and then overlaps with p27Xic1 expression. p27Xic1 expression gradually increases in the CMZ [13]. Gliogenic activity is also gradually activated in the ciliary marginal zone. NM23-X4 inhibits the activity of p27Xic1-mediated gliogenesis at the peripheral side of the p27Xic1-expression domain. This results in proper temporal and spatial regulation of gliogenesis. Single, double and triple asterisks correspond to *P *≤ 0.05, 0.01, and 0.001, respectively; error bars indicate standard error of the mean.

It has been evident that p27Xic1 regulates the cell cycle acting as a cell cycle inhibitor. We therefore examined if the NM23-X4 inhibition of p27Xic1-mediated gliogenesis has any effect on the cell cycle. To address this, we performed a bromodeoxyuridine (BrdU) incorporation assay in the retina and a proliferation assay using early *Xenopus *embryos [13]. Consistent with previous reports [13], p27Xic1 reduces the proliferative potential of cells in both neural retina and early embryos as demonstrated by the decrease of BrdU positive cells (Figure 8A) and the enlargement of blastomeres (Figure 8B,C), respectively. Interestingly, NM23-X4 co-overexpression with p27Xic1 inhibited the p27Xic1-mediated cell cycle inhibition (Figure 8A–C). Furthermore, loss of NM23-X4 function, using *shX4-A *and -*B *constructs, inhibited proliferation in the retina as assayed in cells of the inner nuclear and ganglion cell layer (Figure 8A). These observations suggest that endogenous NM23-X4 functions as an inhibitor of the p27Xic1 cell cycle regulatory action. Overexpression of NM23-X4 or NM23-X1 by itself did not alter proliferation in either retinogenesis or early embryogenesis. This observation may suggest that endogenous NM23-X4 levels are already sufficient to suppress the p27Xic1 activity and overexpression above those levels does not affect the outcome. Further, when we analyzed clone sizes of lipofected cells in *Xenopus *retina, we did not observe any significant changes in the clone size after modulation of NM23-X4 activity (data not shown). This enforces the idea that NM23-X4 primarily acts at the last step of the sequential determination of retinal cell lineage, that is, the production of Müller glia cells without affecting the overall clone proliferation (Figures 5O and 7Q). Last, there was no effect of NM23-X4 on apoptosis in the retina (data not shown). Using a TUNEL assay, we did not observe any change in the ratio of apoptotic cells compared to the control (approximately 3.5% apoptotic cells at stage 33/34).

## Discussion

### Developmental roles of the NM23 family members in retinogenesis

NM23 family members possess NDPK activity, which is required for production of ATP or GTP. In addition, several unique activities of NM23 family members have been reported, such as protein kinase, exonuclease and DNA repair activities [24,25]. Although NM23 family members are expressed in the central nervous system, very little was known about their roles in neural development. We report that all *Xenopus *homologs are expressed in the retina, that NM23-X3 and -X4 are specifically expressed in retinal precursor cells in the CMZ and that NM23-X4 has an essential role in the distribution of retinal cell types through interaction with p27Xic1.

The observation that inhibition of NM23-X4 function by shRNA constructs increased the proportion of Müller cells (Figure 5) indicates that endogenous NM23-X4 functions as a regulator of gliogenesis in the retina. This function is associated with its ability to inhibit p27Xic1-mediated gliogenesis (Figure 6A). Our previous work showed that p27Xic1 plays an important role in both neurogenesis and gliogenesis in a context-dependent manner [8,13,14]. This means that CDKIs regulate the decision between neurogenesis versus gliogenesis depending on the presence of neurogenic stimuli in the progenitors [17]. In the *Xenopus *retina, glial cells are formed after neurogenesis has taken place. The expression of p27Xic1 in the CMZ as retinogenesis progresses is consistent with its role in cell fate determination [13]. As shown in Figure 4D–F, at stage 39, NM23-X4 is expressed at the peripheral side of the CMZ. The expression domain of NM23-X4 overlaps with that of p27Xic1 in the central region of the CMZ (Figure 4F,G). From the expression patterns of NM23-X4 and p27Xic1, their interaction and functions, we propose a model, schematically represented in Figure 8D. According to this, p27Xic1 acts at the central part of the CMZ to induce Müller glial cells. NM23-X4 is responsive to suppress this gliogenic activity of p27Xic1 at the peripheral part of the CMZ, where their two expression domains coincide. This suppression results in inhibition of Müller glial cell production and maintenance of the neurogenic potential of the early progenitors in the retinal cell lineage.

Although it was previously reported that NM23-H4 is largely located in mitochondria [39], our analysis using a deletion construct of NM23-X4 lacking its mitochondria-sorting signal showed that mitochondrial localization is not required for its gliogenic activity (data not shown). This is supported by the observation that all other NM23 members tested showed similar gliogenic activities (Figure 7Q) and p27Xic1 binds to both wild-type NM23-X4 and its amino-terminal processed form (data not shown). It is more likely that the NM23 family regulates the activity of CDKIs in the cytosol because Cip/Kip CDKIs localize in the cytosol and shuttle to the nucleus depending on the cellular context. This notion is also supported by the cytosolic localization observed when we are staining against tagged forms of exogenous NM23-X4 in embryos in addition to the mitochondrial staining (data not shown).

How does NM23-X4 inhibit the gliogenic activity of CDKIs? We showed that direct interaction with the amino-terminal half of p27Xic1 and a specific NM23-X4 activity, probably as a NDPK or protein kinase, are required for the inhibition of p27Xic1. Although several residues of CDKIs are phosphorylated, a majority of the phosphorylation sites are located at the carboxy-terminal half. In the amino-terminal half, only threonine-57 of p21Cip1, serine-10 and tyrosine-88 of p27Kip1 have been reported as phosphorylation sites [40-42]. However, the threonine-57 and serine-10 sites are not conserved in p27Xic1 and, moreover, NM23 family members are not known to possess tyrosine kinase activity, arguing against these sites on p27Xic1 being the ones phosphorylated by NM23-X4. Previously, NM23-H1 was reported to phosphorylate the kinase suppressor of Ras in a histidine dependent manner [29]. Also, the aspartic acid at 319 of aldolase C is phosphorylated by NM23-H1 [43]. In bacteria and plants, histidine kinase activity has very important roles through consequent phosphorylation of aspartic acid and glutamic acid [44,45]. Interestingly, vertebrate CDKIs have three conserved aspartic acids and glutamic acid in the CDK/cyclin binding domain at the amino termini. Our preliminary work has shown that mutation of these residues abrogates the NM23-X4 effect (data not shown). Ongoing work will verify if CDKIs are direct targets of NM23-X4 action.

In addition to the inhibitory role of NM23-X4 on gliogenesis, we have shown that a large gain of NM23-X4 activates gliogenesis. We have provided evidence that, at the endogenous level, NM23-X4 works as a negative regulator of p27Xic1-mediated gliogenesis through direct protein interaction as shown by the knock-down analysis. Data from overexpression assays suggest that NM23-X4 works as an activator of gliogenesis in a mechanism largely independent of p27Xic1. Further work will be required to determine how NM23-X4 may act as an activator of gliogenesis. It is evident that it affects cell fate determination. Recent work has shown that purine-mediated signaling has a major role in eye development [46]. It is of great interest that molecules involved in ATP/ADP enzymatic steps are also part of a regulatory network, along with transcription factors and other partners, to affect eye development. The exact mechanisms are yet to be elucidated in future work.

### NM23-X4-mediated regulation of cell type distribution versus cell cycle regulation

Distribution of retinal cell types can be influenced by modulation of cell cycle regulation and cell death in addition to cell fate determination. Our analysis shows that endogenous NM23-X4 functions as an activator of the cell cycle (Figure 8A–C). This is in accordance with the finding that NM23-X4 binds to the CDK/cyclin domain at the amino-terminal region of p27Xic1. The binding may be antagonistic and compete with the binding of other CDK/cyclin partners, abolishing the formation of a complex. Is this cell cycle related activity responsible for the regulation of the ratio of Müller glial cells in the retina? p27Xic1 has been shown to activate Müller gliogenesis along with its cell cycle inhibitory action. The cell fate determination activity is separable from the cell cycle regulatory one, although both activities are mediated by the amino-terminal region. Our observations regarding binding of NM23-X4 with the amino-terminal region of p27Xic1 and functional assays suggest that NM23-X4 also regulates both the cell fate determination and cell cycle related activity of p27Xic1. Further analysis is required to exclude the possibility of direct cell cycle involvement in the change of cell type distribution.

### Diverse roles of CDKIs and NM23s in biological processes

As mentioned, the amino termini of CDKIs regulate both cell cycle and cell fate determination. Recent papers show that the carboxyl termini of CDKIs directly bind with regulators of cytoskeletal proteins. For example, p21Cip1, p27Kip1, and p57Kip2 directly bind to Rho kinase/ROCK, RhoA and Stathmin, and LIM kinase, respectively [18-22], to influence neurite formation and cell migration. Knockout mice of CDKIs show normal cell division, suggesting that they are not essential for the fundamental cell cycle regulation. Furthermore, differentiation factors are known to induce expression of CDKIs. Taken together, these observations indicate that the Cip/Kip CDKIs have three independent functions: cell cycle inhibition, cell fate determination, and differentiation. It is demonstrated that, upon induction by secreted differentiation factors, the primary role of CDKIs lies in their ability to regulate the process of neuro/gliogenesis by co-coordinating cell cycle arrest, cell fate determination, and differentiation. Our results also demonstrate that NM23 members can work in a CDKI-dependent context and participate in this regulatory network. NM23-H1 was originally identified as an inhibitor of cancer metastasis [24]. However, still little is known about the mechanism of this action. Recently, some papers showed that the Cip/Kip CDKIs are major regulators of cancer metastasis [19]. Our observations overall propose the attractive hypothesis that CDKIs, regulated by NM23s, may have a major role in controlling both neural development and cancer metastasis.

## Conclusion

We have identified NM23-X4 as a binding partner of p27Xic1 and showed that its expression in the CMZ overlaps with that of p27Xic1. Our functional analysis demonstrates that endogenous NM23-X4 negatively regulates p27Xic1-mediated gliogenesis and potentiates neurogenesis through protein-protein interactions. The glia-inhibitory function of NM23-X4 is likely to require its NDPK or histidine dependent kinase activity. Our results suggest that NM23-X4 functions in a temporal and spatial manner to regulate the process of gliogenesis during sequential retinal cell fate determination.

## Materials and methods

### Bacterial two-hybrid screening and plasmid construction

The bacterial two-hybrid screening was performed by BacterioMatch™ (Stratagene Texas, USA) using a bait construct, *pBT-Xic1*, that has the amino terminus (1-96 amino acids) of p27Xic1 and the BacterioMatch *Xenopus *embryo library (Stratagene). A positive clone including NM23-X4 was identified from several positive clones after confirmation steps. NM23-X1, -X2, and -X3 expression plasmids were kindly provided by Dr A Mazabraud [27]. Through a *Xenopus *EST database search and blast search we identified *NM23-X4 *[GenBank:BU900096], -*X5 *[GenBank:CK805921], -*X6 *[GenBank:BX845338], -*X7 *[GenBank:BG555939] and -*X8 *[GenBank:BE679366]. All clones were obtained and subcloned in a *pCS2FLAG *vector. Mutants H148C, S150G, ΔKPN loop, and Δ(1-33) of NM23X4 were made with a site-directed mutagenesis or deletion strategy in *pCS2FLAG *according to standard procedures. The p27Xic1, its mutants and the p16Xic2 constructs were described previously [13,30]. HA-tagged constructs were made using *HA-pcDNA3 *vector.

### Design of shRNA constructs for p27Xic1 and NM23-X4

shRNA target sequences were designed using the web site siDirect [47]. Two shRNA constructs against different target sites were constructed using *pSUPER *(Oligoengine Seattle, Washington, USA)). The designed oligonucleotides for p27Xic1 and NM23-X4 are as follows: *shXic1-A *forward, gatccccgcggtgccagggagttaaattcaagagatttaactccctggcaccgctttttggaaa; *shXic1-A *reverse, agcttttccaaaaagcggtgccagggagttaaatctcttgaatttaactccctggcaccgcggg; *shXic1-B *forward, gatccccccgtggagactaaagatgtttcaagagaacatctttagtctcccacggtttttggaaa; *shXic1-B *reverse, agcttttccaaaaaccgtggagactaaagatgttctcttgaaacatctttagtctccacggggg; *shX4-A *forward, gatcccccgactggtgggagaaatcattcaagagatgatttctcccacagtcgtttttggaaa; *shX4-A *reverse, agcttttccaaaaacgactggtgggagaaatcatctcttgaatgatttctcccaccagtcggggg; *shX4-B *forward, gatccccgcagcgtggcttcacattattcaagagataatgtgaagccacgctgctttttggaaa; *shX4-B *reverse, agcttttccaaaaagcagcgtggcttcacttatctcttgaataatgtgaagccacgctgcggg. The oligonucleotides were annealed and then ligated in *pSUPER*.

### *Xenopus *embryo manipulation, mRNA injection, and lipofection

*Xenopus laevis *embryos were obtained by *in vitro *fertilization, and staged according to Niewkoop and Faber. All embryo manipulations were in accordance with the UK Home Office regulations and guidelines. For mRNA injection, all plasmids (*pCS2FLAG-NM23X1*, *X4*, *pCS2-p27Xic1*, *pCS2-nβgal*) were linearized and transcribed *in vitro *using the mMessage mMachine kit (Ambion Austin, TX, USA)). The mRNAs were injected into one blastomere at the two-cell stage. Lipofection into eye primordia was performed as described [48]. The indicated constructs were lipofected in the eye field of embryos at stage 15. At stage 41, the lipofected embryos were fixed and cryostat sectioned as previously described. For cell-tracing purposes, *pGFP *plasmid was co-lipofected with all plasmids. In some cases, co-lipofection was confirmed by immunohistochemistry against the myc or FLAG tag. Cell types were determined by morphology and location of cell body and nucleus along the retinal epithelium and confirmed by cell type specific antibody staining. About 400 cells from 6 retinas were counted in each condition. Each experiment was repeated at least three times. Statistical analysis was performed by one-way ANOVA.

### Proliferation assays

BrdU experiments in *Xenopus *retina were performed as described in our previous paper [13]. Briefly, the indicated construct was co-lipofected with a GFP plasmid at stage 15. BrdU was injected in the abdomen from stage 30 to stage 41 at intervals of 8 h. The embryos were then fixed in 4% paraformaldehyde and sectioned. The section was treated with 2 N HCl and subjected to BrdU antibody staining according to the manufacturer's protocol (Roche Lewes. East Sussex, UK)). We then determined the ratios of BrdU and GFP double positive cells of the inner nuclear layer (Müller glial cells, bipolar, amacrine and horizontal cells) and ganglion cell layer. For the proliferation assay in early embryos, mRNA was injected into a right blastomere at the two-cell stage and the injected embryos were fixed at stage 7. The cell diameter of approximately 20 cells (longest length) was measured after taking images using the Openlab software (Improvision, Coventry, West Midland, UK). Then, the ratio between the diameter of injected and uninjected sides was calculated (Figure 8C).

### RNA extraction and RT-PCR

RNA extraction and RT-PCR were carried out as described [49]. The primers used were: NM23-X1 (forward, 5'-GAGGGACTGAATGTGGTAAA-3'; reverse, 5'-TTTAAACCACAAGGCAATTT-3'); NM23-X4 (forward, 5'-AGCAGAACATTACCATGACC-3'; reverse, 5'-TAGCCTGGGAAGAGTCTGTA-3'). The primers for ornithine decarboxylase have been described previously [30].

### *In situ *hybridization and immunohistochemistry

*In situ *hybridization of whole embryos and sections was carried out as described previously [13]. Probes were prepared after linearization of pBluescript plasmids encoding the full-length cDNA of NM23 members or p27Xic1 and digoxigenin- or FITC-labelling *in vitro *with polymerase T7 or T3. *In situ *hybridization on cryostat sections was performed after untreated embryos were fixed, sucrose treated and embedded in OCT compound. Colour reactions were performed using BM Purple and Fast Red (Roche) according to standard procedures. Immunohistochemistry was preformed as described [8]. Primary antibodies used were anti-R5 (1:1), anti-CRALBP (1:1000; from J Saari), anti-calbindin (1:100; Sigma St. Louis, MO, USA), anti-39.4 D5 (1:100), and anti-FLAG (1:100; Sigma). All immunostainings were visualized with fluorescent conjugated secondary antibodies under a fluorescent microscope.

### Cell culture, immunoprecipitation, and western blotting

COS-7 cells were cultured in DME media/Glutamax (Invitrogen, Paisley, Renfrewshire, UK) containing 10% fetal bovine serum, 100 U/ml penicillin, and 100 μg/ml streptomycin. Immunoprecipitation was carried out as described [49]. Briefly, about 100,000 COS-7 cells/well were transfected using Lipofectamin 2000 (Invitrogen See above) according to the manufacturer's protocol. After 20 h, MG132 (final concentration 50 μM) or AMP-PNP (200 μM) was added to the medium. Then, 4 h later the cells were washed several times with 1× phosphate-buffered saline and lysed in 500 μL of lysis buffer (20 mM Tris-HCl (pH 7.5), 150 mM NaCl, 1% Nonidet P-40, 100 μM PMSF). The supernatants were isolated by centrifugation. We performed immunoprecipitation using about 90% of the cell lysate of COS-7 cells after measurement of the protein level and used about 10% of the lysate (20 μg of protein) for confirmation of levels of expressed proteins. For immunoprecipitation, the lysates were incubated with 2 μg of anti-FLAG M2 (Sigma) for 2 h at 4°C. Then, 20 μL of Protein G-sephrose (Sigma) was added to the mixture. The mixtures were incubated for 1 h at 4°C. After several washes by 1× phosphate-buffered saline, proteins were eluted from the sepharose with 1× SDS buffer. The obtained proteins were separated in 8–15% SDS-PAGE, blotted on the PVDF membrane (Immobilon-P, GE Healthcare, Little Chalfont

Buckinghamshire UK), and detected with chemiluminescence (GE Healthcare)). For detection, we used anti-FLAG M2 (Sigma) for FLAG-tagged protein, and 3F10 (Roche) for HA-tagged proteins. (Note that in the immunoprecipitation experiments, although the expression levels of NM23-X3, NM23-X6, NM23-H4, and p17Xic3 are lower than other proteins, these proteins resulted in significant amounts of immunoprecipitated proteins. We used about 10% of the lysate for checking the expression level and about 90% for immunoprecipitation, suggesting that the amounts of proteins in the immunoprecipitation were sufficient for the binding.)

## Abbreviations

BrdU: bromodeoxyuridine; CDK: cyclin dependent kinase; CDKI: cyclin dependent kinase inhibitor; CMZ: ciliary marginal zone; GFP: green fluorescent protein; NDPK: nucleotide diphosphate kinase; shRNA: short hairpin RNA.

## Competing interests

The authors declare that they have no competing interests.

## Authors' contributions

SO, TM, and AB designed and performed the majority of experiments. CTW provided technical assistance and KH contributed to the two-hybrid screening. MZ provided reagents and initiation discussions. SO and AB wrote the manuscript. All authors read and approved the manuscript.
